# Evidence-based guideline: premature ovarian insufficiency^†^^,^^‡^

**DOI:** 10.1093/hropen/hoae065

**Published:** 2024-12-09

**Authors:** Nick Panay, Richard A Anderson, Amy Bennie, Marcelle Cedars, Melanie Davies, Carolyn Ee, Claus H Gravholt, Sophia Kalantaridou, Amanda Kallen, Kimberly Q Kim, Micheline Misrahi, Aya Mousa, Rossella E Nappi, Walter A Rocca, Xiangyan Ruan, Helena Teede, Nathalie Vermeulen, Elinor Vogt, Amanda J Vincent, ESHRE, ASRM, CREWHIRL, and IMS Guideline Group on POI, Ladan Yeganeh, Rinky Giri, Madeline Flanagan

**Affiliations:** Queen Charlotte’s and Chelsea Hospital, Imperial College London, London, UK; Centre for Reproductive Health, Institute for Regeneration and Repair, University of Edinburgh, Edinburgh, UK; Daisy Network Charity, Suffolk, UK; Department of Obstetrics, Gynecology and Reproductive Sciences, University of California, San Francisco, School of Medicine, San Francisco, CA, USA; Reproductive Medicine Unit, University College London Hospital, London, UK; NICM Health Research Institute, Western Sydney University, Penrith, NSW, Australia; Department of Endocrinology, Aarhus University Hospital, Aarhus, Denmark; Third Department of Obstetrics and Gynecology, National and Kapodistrian University of Athens, Athens, Greece; Division of Reproductive Endocrinology and Infertility, University of Vermont Larner College of Medicine, Burlington, VT, USA; Department of Obstetrics, Gynecology and Reproductive Sciences, Yale School of Medicine, New Haven, CT, USA; RESOLVE: The National Infertility Association, McLean, VA, USA; Faculté de Médecine, Hôpital Bicêtre, Université Paris Saclay, Le Kremlin Bicêtre, France; Monash Centre for Health Research and Implementation (MCHRI), Monash University, Clayton, Australia; Department of Clinical, Surgical, Diagnostic and Pediatric Sciences, University of Pavia, Pavia, Italy; Research Center for Reproductive Medicine, Gynecological Endocrinology and Menopause, IRCCS San Matteo Foundation, Pavia, Italy; Department of Neurology, Mayo Clinic, Rochester, MN, USA; Department of Gynecological Endocrinology, Beijing Obstetrics and Gynecology Hospital, Beijing, China; Monash Centre for Health Research and Implementation (MCHRI), Monash University, Clayton, Australia; ESHRE Central Office, Strombeek-Bever, Belgium; Department of Medicine, Haukeland University Hospital, Bergen, Norway; Monash Centre for Health Research and Implementation (MCHRI), Monash University, Clayton, Australia

**Keywords:** premature ovarian insufficiency, primary ovarian insufficiency, hormone therapy, hormone replacement therapy, combined oral contraceptive, puberty induction, fertility, bone, brain, cardiovascular

## Abstract

**STUDY QUESTION:**

How should premature/primary ovarian insufficiency (POI) be diagnosed and managed based on the best available evidence from published literature?

**SUMMARY ANSWER:**

The current guideline provides 145 recommendations on symptoms, diagnosis, causation, sequelae, and treatment of POI.

**WHAT IS KNOWN ALREADY:**

Premature ovarian insufficiency (POI) presents a significant challenge to women’s health, with far-reaching implications, both physically and emotionally. The potential implications include adverse effects on quality of life; fertility; and bone, cardiovascular, and cognitive health. Although hormone therapy (HT) can mitigate some of these effects, many questions still remain regarding the optimal management of POI.

**STUDY DESIGN, SIZE, DURATION:**

The guideline was developed according to the structured methodology for development of ESHRE guidelines. Key questions were determined by a group of experts and informed by a scoping survey of women and health care professionals. Literature searches and assessments were then performed. Papers published up to 30 January 2024 and written in English were included in the guideline. An integrity review was conducted for the randomized controlled trials (RCTs) on POI included in the guideline.

**PARTICIPANTS/MATERIALS, SETTING, METHODS:**

Based on the collected evidence, recommendations were formulated and discussed within the guideline development group until consensus was reached. Women with lived experience of POI informed the recommendations in general, and particularly on those on provision of care. A stakeholder review was organized after finalization of the draft. The final version was approved by the guideline development group and the ESHRE Executive Committee.

**MAIN RESULTS AND THE ROLE OF CHANCE:**

New data indicate a higher prevalence of POI, 3.5%, than was previously thought. This guideline aims to help health care professionals to apply best practice care for women with POI. The recent update of the POI guideline covers 40 clinical questions on diagnosis of the condition, the different sequelae, including bone, cardiovascular, neurological and sexual function, fertility and general well-being, and treatment options, including HT. The list of clinical questions was expanded from the previous iteration of the guideline (2015) based on the scoping survey and appreciation of emerging knowledge of POI. Questions were added on the role of anti-Müllerian hormone (AMH) in the diagnosis of POI, fertility preservation, muscle health, and specific considerations for HT in iatrogenic POI. Additionally, the topic on complementary treatments was extended with specific focus on non-hormonal treatments and lifestyle management options. Significant changes from the previous 2015 guideline include the recommendations that only one elevated FSH >25 IU is required for diagnosis of POI, and guidance that AMH testing, repeat FSH measurement, and/or AMH may be required where there is diagnostic uncertainty. Recommendations were also updated regarding genetic testing, estrogen doses and regimens, use of the combined oral contraceptive and testosterone therapy. Women with lived experience of POI informed the recommendations on provision of care.

**LIMITATIONS, REASONS FOR CAUTION:**

The guideline describes different management options, but it must be acknowledged that for most of these options, supporting evidence is limited for POI.

**WIDER IMPLICATIONS OF THE FINDINGS:**

The guideline provides health care professionals with clear advice on best practice in POI care, based on the best evidence currently available. In addition, a list of research recommendations is provided to guide further studies in POI.

**STUDY FUNDING/COMPETING INTEREST(S):**

The guideline was developed and funded by ESHRE, American Society for Reproductive Medicine (ASRM), Centre for Research Excellence in Women's Health in Reproduction Life (CRE-WHiRL), and International Menopause Society (IMS), covering expenses associated with the guideline meetings, literature searches, and dissemination of the guideline. The guideline group members did not receive payments. N.P. declared grants from Bayer Pharma (research and consultancy) and NIHR—research POISE; consulting fees from Abbott, Astellas, Bayer, Besins, Lawley, Mithra, Theramex, Viatris; honoraria from Astellas, Bayer, Besins, Gedeon Richter, Theramex, Viatris; support for attending meetings and/or travel from Astellas, Bayer, Theramex, Viatris; President, International Menopause Society, Medical Advisory Committee member, British Menopause Society, Patron Daisy Network. A.J.V. declared grants from Amgen Australia, Australian NHMRC, and Australian MRFF; consulting fees from IQ Fertility; honoraria from the Australasian Menopause Society; participation on a Data Safety Monitoring Board or Advisory Board of Astellas; Board Member of the International Menopause Society (2020 to current) and Past president of the Australasian Menopause Society (2017–2019); R.A.A. declared grants from Roche (Research support, to institution), and participation on a Data Safety Monitoring Board of Bayer. M.C. declared grants from NHI; payments or honoraria from Up-to-Date (as editor/reviewer); Board Member of American Society of Reproductive Medicine, and of American Gynecological and Obstetrical Society. M.D. declared (NIHR—HTA Reference Number: NIHR133461; NIHR—HTA Reference Number: NIHR128757; Action Medical Research and Borne: GN2818) consulting fees from a small personal medical practice, support for attending meetings and/or travel from ESHRE, Bayer and UCLH special Trustees; Participation on the Advisory Board of the British Menopause Society, UKSTORE project, the Progress Educational Trust, and the Turner Syndrome Support Society UK; Leadership or fiduciary roles in the British Fertility Society (Trustee), Elizabeth Garrett Anderson Hospital Charity (chair of Trustees), and the Essex Wynter charitable trust (Trustee). C.E. declared being Chair of a SIG from the Royal Australian College of General Practitioners Integrative Medicine Specific Interest Group and Program Lead for Next Practice Western Sydney Integrative Health. C.H.G. declared grants from Novo Nordisk Foundation (Nos. NNF15OC0016474 and NNF20OC0060610), sygesikringen danmark (No 2022-0189), and the Independent Research Fund Denmark (Nos. 0134-00406 and 0134-00130B); consulting fees from Novo Nordisk, Merck, and Astra Zeneca. S.K. declared grants from Roche diagnostics. A.K. declared grants from NIH R01 5R01HD101475; consulting fees as Medical Reviewer for Flo and for Healthline; honoraria as Medical Consultant for Summus; support for attending meetings from the Reproductive Scientist Development Program; Society for Reproductive Investigation Council Member and Society for Assisted Reproduction Registry/Validation Chair; R.E.N. declared consulting fees from Astellas, Bayer Pharma, Besins Healthcare, Fidia, Theramex; honoraria from Abbott, Astellas, Exeltis, Fidia, Gedeon Richter, Merck & Co, Novo Nordisk, Shionogi Limited, Theramex, Viatris; payment for expert testimony from Vichy Laboratories; Participation in Data Safety Monitoring Board of Advisory board from Astellas and Bayer Healthcare; President elect of the International Menopause Society (IMS). H.T. declared a grant from NHMRC Centre for Research Excellence for women’s health in reproductive life. A.B. declared being chair of the Daisy Network Charity. The other authors have no conflicts of interest to declare.

**DISCLAIMER:**

*This guideline represents the views of ESHRE, ASRM, CRE-WHiRL, and IMS, which were achieved after careful consideration of the scientific evidence available at the time of preparation. In the absence of scientific evidence on certain aspects, a consensus between the relevant stakeholders has been obtained. Adherence to these clinical practice guidelines does not guarantee a successful or specific outcome, nor does it establish a standard of care. Clinical practice guidelines do not replace the need for application of clinical judgement to each individual presentation, nor variations based on locality and facility type*.

*The collaborating societies make no warranty, expressed or implied, regarding the clinical practice guidelines and specifically exclude any warranties of merchantability and fitness for a particular use or purpose*. *(Full disclaimer available at www.eshre.eu/guidelines*.*)*

WHAT DOES THIS MEAN FOR PATIENTS? Informed by those with lived experience of premature ovarian insufficiency (often shortened to POI), in addition to current evidence, this guideline aims to facilitate prompt diagnosis of POI, conveyed in a sensitive manner, and shared decision making for personalized best practice management. This will assist in effectively addressing recognized patient dissatisfaction, care variation, non-adherence with therapy, and resultant poorer outcomes in women with POI.

## Introduction

This guideline on premature ovarian insufficiency (POI) offers best practice advice on the care of women with POI.

POI is a clinical condition characterized by loss of ovarian function, indicated by irregular menstrual cycles together with biochemical confirmation of ovarian insufficiency before the age of 40. POI is to be differentiated from the usual-age of menopause, as women with POI have unique needs and management options. They may not only suffer from symptoms associated with estrogen deficiency, but can also experience other issues, with a significant impact on their quality of life and later health outcomes. POI affects fertility, bone health, cardiovascular health, sexual function, psychological health, and neurological function, making it a challenge for patients and health care professionals (HCPs) (Webber *et al.*, 2016).

This guideline on POI describes the impact of POI on these different domains and discusses treatment options for each of them and monitoring needs where relevant. The information on treatment indications is included in a chapter on hormone therapy (HT), which also covers further topics related to risks and options for HT in general and in women with POI, and comorbidities where data exist. In other chapters, non-hormonal and complementary treatments in POI are also discussed, as well as lifestyle and puberty induction.

Furthermore, the clinical guideline provides recommendations on the diagnosis of POI and the recommended assessment of causation, with some elaborated guidance on care for women at the time of diagnosis and implications for their relatives.

This paper summarizes the recommendations as they are included in the Evidence-based Guideline on POI. For further information and details, the reader is referred to the full guideline published on the societies’ websites.

This guideline is limited to POI and does not apply to women with low ovarian reserve. Reference to early menopause is included where evidence is available but was not the focus of the key questions.

## Materials and methods

The guideline was developed according to a well-documented methodology that is universal to ESHRE guidelines (Vermeulen *et al*., 2020). The guideline development group (GDG) was composed of past members of the guideline group from 2015 and additional experts, also representing the collaborating societies, constituting an international group of experts. The guideline group included two patient representatives/advocates.

Key questions were formulated by the guideline group, based on the list of key questions from 2015, but extended following a scoping survey amongst patients and health professionals. The final guideline was built from a list of 40 key questions, of which four were answered with narrative reviews (hereafter referred to as ‘key questions’) and 36 with systematic reviews as PICO (Patient, Intervention, Comparison, Outcome) questions. For each PICO question, databases (PUBMED/MEDLINE) were searched from inception up to 30 January 2024, and limited to studies written in English. From the literature searches, studies were selected based on the PICO questions, assessed for quality, and summarized in evidence tables (www.eshre.eu/guidelines). For the narrative questions, a similar literature search was conducted. Collected data were summarized in a narrative summary and conclusions were formulated.

An integrity review using the Research Integrity in Guidelines and evIDence synthesis (RIGID) methodology was performed on 32 randomized controlled trials (RCTs) of treatments in the POI-specific population (Mousa *et al.*, 2024). GDG meetings were organized (primarily online), for presentation and discussion of the evidence and draft recommendations until consensus was reached. Each recommendation was labelled as strong or conditional, and the Grading of Recommendations, Assessment, Development and Evaluations (GRADE) approach was applied to indicate the strength of the supporting evidence (High ⊕⊕⊕⊕, Moderate ⊕⊕⊕◯, Low ⊕⊕◯◯, Very low ⊕◯◯◯). Good practice points (GPPs) based on clinical expertise were added where relevant to clarify the recommendations or to provide further practical guidance.

**Figure 1.**
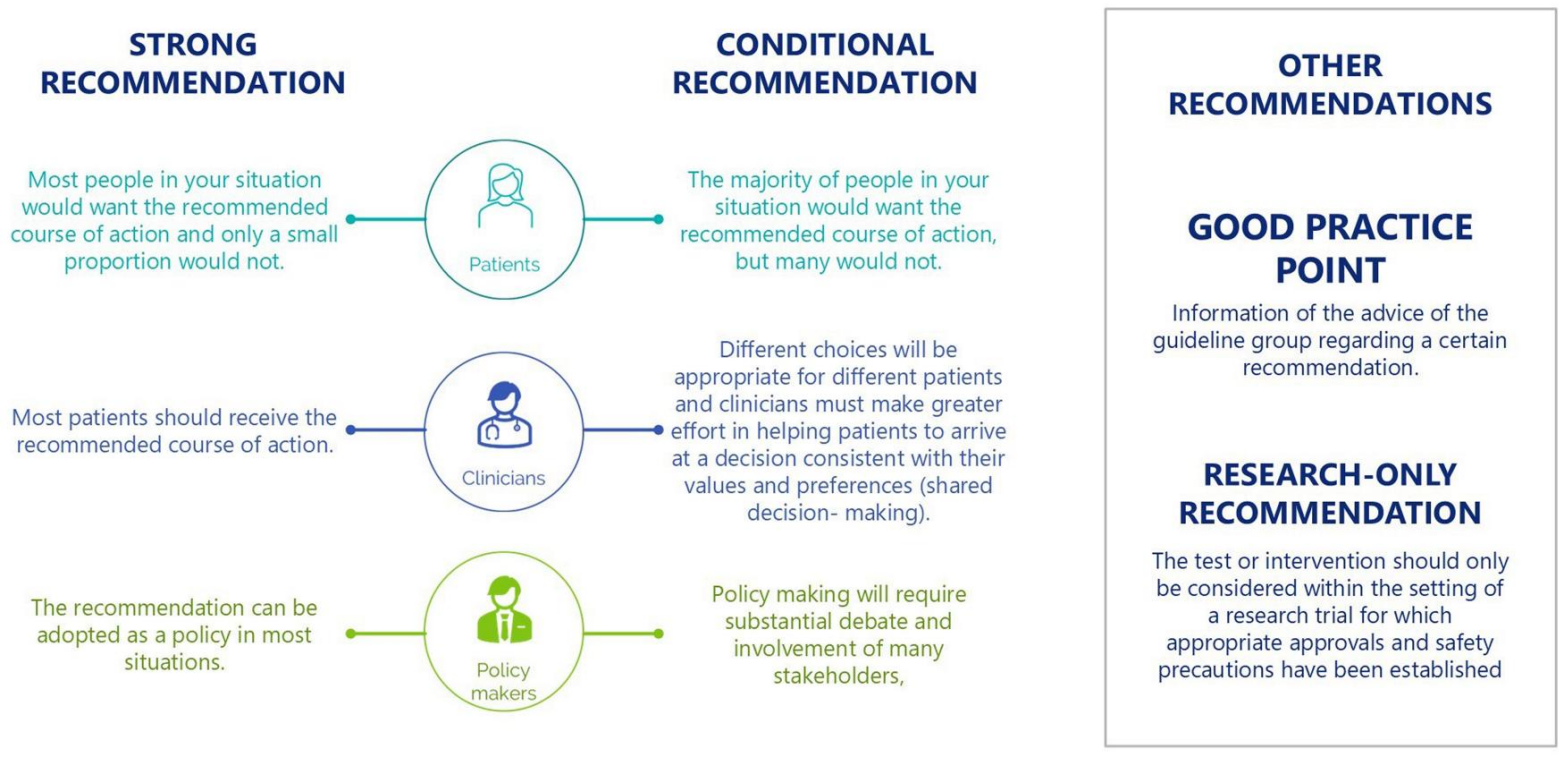
Suggested interpretation of the strong and conditional recommendations included in the guideline by patients, health care professionals (HCPs) and health care policy makers.

Strong recommendations suggest that the recommended option applies in most circumstances, whereas conditional recommendations are dependent on specific factors, which need to be considered with benefits/risks weighed before applying a given option (Fig. 1).

The guideline draft and an invitation to participate in the stakeholder review (i.e. public consultation) were published on the ESHRE website between 17 April and 27 May 2024. The invitation to contribute to the stakeholder review was circulated to all collaborating and partnering organizations. All comments were processed by the guideline group, either by adapting the content of the guideline and/or by replying to the reviewer. The review process was summarized in the review report, which is published on the ESHRE website (www.eshre.eu/Guidelines). Overall, 61.0% of the 374 comments to the content resulted in an adaptation or correction in the guideline text.

This guideline will be considered for update 4 years after publication, with an intermediate assessment of the need for updating 2 years after publication.

## Results (recommendations)

The scope of the guideline on POI is to provide guidance on the management of POI. In line with research on the topic, terminology, and discussion, the guideline is focused on women. The guideline group recognizes that there are individuals living with POI who are transgender or who do not identify with the terms used in the literature. Throughout, the term ‘women with POI’ is used, but this is not intended to isolate, exclude, or diminish any individual’s experience nor to discriminate against any group.

### Introduction to POI

**Key Question: What should this condition be called?**

**Table:** 

The guideline group recommends that the term ‘premature ovarian insufficiency’ is used to describe this condition in research and clinical practice.	GPP

**Key Question: How should POI be defined?**

**Table:** 

Premature ovarian insufficiency (POI) is a condition defined by loss of ovarian activity before the age of 40 years. POI is characterized by amenorrhea or irregular menstrual cycles with elevated gonadotropins and low estradiol. In this guideline, cessation of ovarian function in women aged from 40 and <45 (age 40–44 years) will be termed early menopause. Early menopause is outside the scope of the current guideline, but the evidence and recommendations may be relevant to women with early menopause.	GDG STATEMENT

**Key Question: What is the prevalence of POI in the general population?**

**Table:** 

The reported prevalence of non-iatrogenic POI varies from ∼1% in older studies to 3.5% in recent publications. Population characteristics such as ethnicity may affect the prevalence of non-iatrogenic POI.	GDG STATEMENT

**PICO Question: What are the risk factors for POI?**

**Table:** 

The guideline group recommends that in view of the long-term health consequences of POI, efforts should be made to reduce the risk of POI. Modifiable factors may include: gynaecological surgical practice lifestyle factors such as smoking treatment regimens for malignant and chronic diseases.	GPP
The guideline group recommends that women with risk factors for POI are identified and counselled regarding POI risk and fertility preservation.	GPP

**Figure 2.**
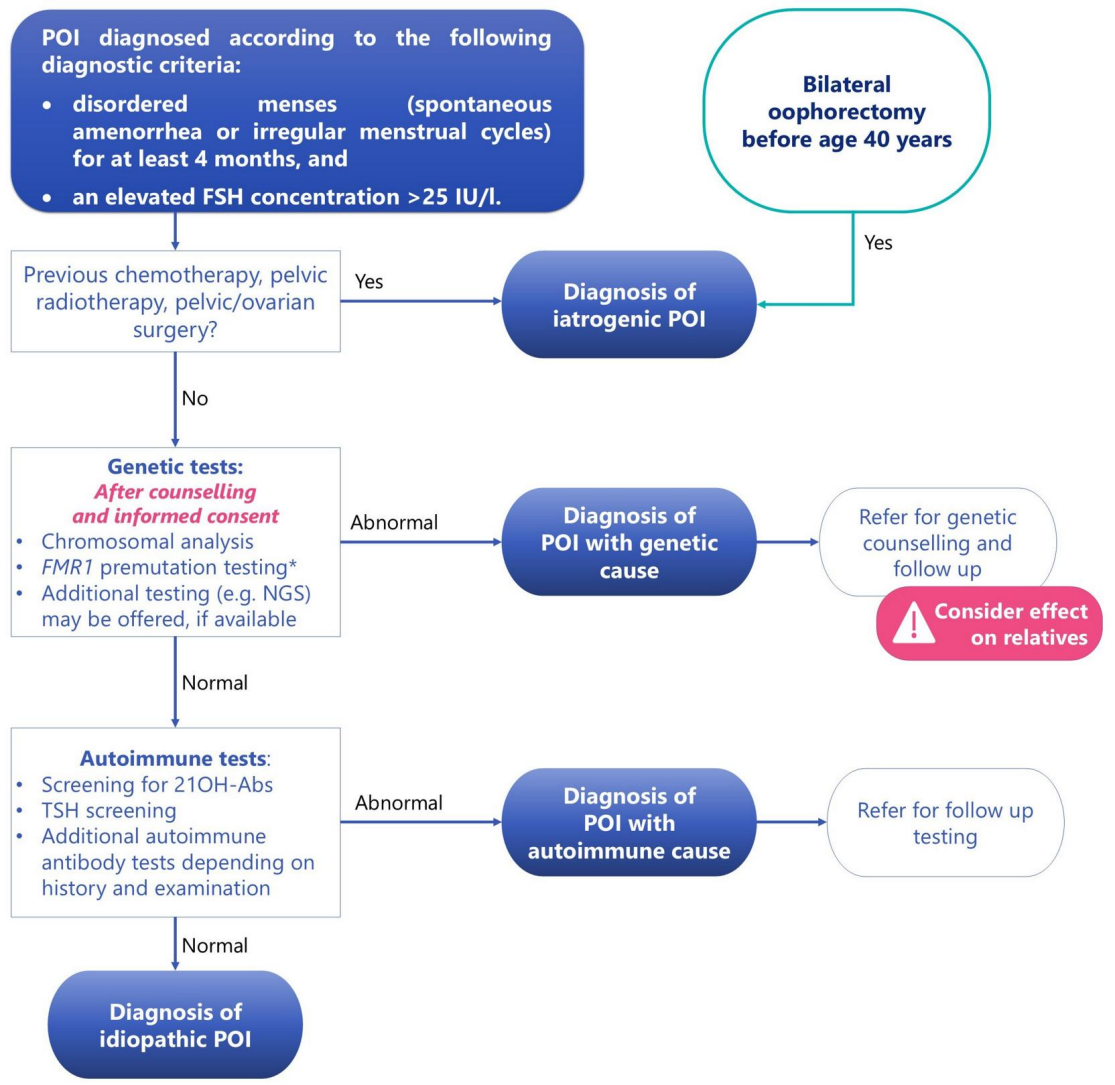
**Summary of the recommendations on diagnosis of premature ovarian insufficiency (POI), as well as the recommended further testing to establish a cause for POI**. *Fragile X premutation testing is indicated in all women diagnosed with POI. This needs to be performed as a specific test as multigene panels and NGS are not useful in detecting FMR1 premutation. 21OH-Abs, 21-hydroxylase autoantibodies; BSO, bilateral salpingo-oophorectomy; NGS, next-generation sequencing; TSH, thyroid-stimulating hormone.

Diagnosis of POI (Fig. 2)

**PICO Question: What are the symptoms of POI?**

**Table:** 

The guideline group recommends that health care professionals (HCPs) enquire about symptoms of estrogen deficiency in women presenting with irregular menstrual cycles or amenorrhea.	GPP
The guideline group recommends HCPs consider and exclude the diagnosis of POI in women aged <40 years who have amenorrhea/irregular menstrual cycles or estrogen-deficiency symptoms.	GPP

**PICO Question: What investigations should be performed for diagnosis of POI?**

**Table:** 

HCPs should diagnose POI based on the presence of spontaneous amenorrhea or irregular menstrual cycles and biochemical confirmation.	STRONG **⊕⊕◯◯**
The guideline group recommends the following diagnostic criteria: disordered menstrual cycles (spontaneous amenorrhea or irregular menstrual cycles) for at least 4 months and an elevated FSH concentration > 25 IU/l. FSH assessment should be repeated after 4–6 weeks if there is diagnostic uncertainty. FSH testing for the diagnosis of POI does not have to be timed to a specific day of the menstrual cycle.	GPP
The guideline group recommends that HCPs consider these points when diagnosing POI: Pregnancy should be excluded in women presenting with amenorrhea. Use of hormonal therapy (including oral, injectable, or long-acting contraceptives) may conceal or cause amenorrhea or irregular menstrual cycles, and potentially lower FSH concentrations. Some hormonal therapy (e.g. combined oral contraceptive) may need to be ceased before a diagnosis of POI can be confirmed. Women who had bilateral salpingo-oophorectomy (BSO) before age 40 have a diagnosis of POI, and additional diagnostic testing is unnecessary.	GPP
The guideline group does not recommend diagnosing POI based on serum estradiol concentrations. However, a low estradiol concentration indicates hypoestrogenism, and in combination with an elevated FSH concentration, provides additional confirmation of the POI diagnosis.	GPP

**PICO Question: What is the role of anti-Müllerian hormone to predict/diagnose POI?**

**Table:** 

Anti-Müllerian hormone (AMH) should not be used as the primary diagnostic test for POI.	STRONG **⊕◯◯◯**
The guideline group recommends that AMH testing may be useful to confirm POI diagnosis where FSH results are inconclusive, but AMH results need to be interpreted within the clinical context.	GPP
The guideline group recommends that HCPs do not routinely perform AMH testing to predict POI due to insufficient evidence of accuracy.	GPP

**PICO Question: What are the known causes of non-iatrogenic POI and how should they be investigated?**

**Table:** 

The guideline group recommends that HCPs inform women with POI of the different causes of POI, the limitations of current knowledge and testing for causes of POI, and that an exact cause may not be identified.	GPP
The guideline group recommends that HCPs discuss the risk of POI as part of the consent process before a medical or surgical intervention that may cause POI.	GPP
The guideline group recommends that HCPs discuss the implications of genetic testing before the test is performed. Referral for comprehensive genetic counselling should be considered.	GPP
Chromosomal analysis testing is recommended for all women with non-iatrogenic POI.	STRONG **⊕⊕◯◯**
*FMR1* premutation (Fragile X syndrome gene) testing is recommended for all women with non-iatrogenic POI	STRONG **⊕⊕◯◯**
Where available and after comprehensive genetic counselling, additional genetic testing (e.g. next-generation sequencing) can be offered to all women with non-iatrogenic POI to identify other potential genes that may cause POI	CONDITIONAL **⊕⊕◯◯**
The guideline group recommends that the age of a woman with POI should not be used to restrict access to genetic testing.	GPP
Screening for 21-hydroxylase autoantibodies (21OH-Abs) should be performed in women with POI of unknown cause.	STRONG **⊕◯◯◯**
Screening for anti-ovarian autoantibodies should not be used to diagnose autoimmune POI.	STRONG **⊕◯◯◯**
Thyroid function should be assessed by measuring thyroid-stimulating hormone (TSH) at POI diagnosis. TSH measurement should be repeated every 5 years or when symptoms arise.	STRONG **⊕◯◯◯**
The guideline group recommends that HCPs do not routinely perform thyroid peroxidase (TPO) antibody screening as part of testing for autoimmune causes of POI due to the high prevalence of positive TPO antibodies in the general community.	GPP

**PICO Question: how often should tests for autoantibodies be repeated?**

**Table:** 

Women with POI and positive 21OH-Abs should be referred to an endocrinologist for testing of adrenal function.	STRONG **⊕◯◯◯**
If 21OH-Abs are negative in women with POI, there is no indication for re-testing later in life, unless signs or symptoms of adrenal insufficiency develop.	STRONG **⊕◯◯◯**
Women with POI with abnormal TSH levels should be evaluated and treated for thyroid hormone disorders.	STRONG **⊕◯◯◯**

**Care for women with POI at diagnosis**

**Table:** 

The guideline group recommends that HCPs convey the diagnosis of POI in a compassionate and sensitive manner, provide personalized evidence-based information about the condition, and ensure time for the women to ask questions.	GPP
The guideline group recommends shared decision making and support for continuity of care in managing POI.	GPP
The guideline group recommends referral of women with POI to appropriate support groups and mental health care.	GPP

**Key Question: What are the possible implications for relatives of women with POI?**

**Table:** 

The guideline group recommends that relatives of women with the *FMR1* premutation or other identified genetic causes of POI should be offered genetic counselling and testing.	GPP
Female relatives (such as sisters or daughters) of women with non-iatrogenic POI should be counselled that they are at increased risk of developing POI themselves.	STRONG **⊕⊕◯◯**
The guideline group recommends that female relatives (such as sisters or daughters) of women with non-iatrogenic POI are offered support regarding their increased risk of POI, and ovarian reserve testing may be helpful.	GPP
The guideline group recommends that female relatives (such as sisters or daughters) of women with non-iatrogenic POI should be informed of the signs and symptoms of POI and should promptly seek medical advice if this occurs.	GPP
The guideline group recommends that female relatives (such as sisters or daughters) of women with non-iatrogenic POI should be informed that there are no established methods for predicting or preventing POI. Some relatives may wish to consider family planning and fertility preservation options.	GPP

### Sequelae of POI

**PICO Question: What are the consequences of POI for life expectancy?**

**Table:** 

Women with POI should be informed that POI without HT is associated with reduced life expectancy, largely due to cardiovascular disease.	STRONG **⊕⊕◯◯**
HT is recommended for women with POI until the usual age of menopause for primary prevention to reduce the risk of morbidity and mortality, whether there are estrogen deficiency symptoms or not.	STRONG **⊕◯◯◯**
The guideline group recommends that women with POI should be encouraged to adopt a healthy lifestyle (including avoiding smoking, having a healthy diet and regular physical activity, and maintaining a healthy weight range) to reduce cardiovascular risk.	GPP

**PICO Question: What are the consequences of POI for fertility?**

**Table:** 

Women with POI should be informed that POI substantially reduces the chances of natural conception.	STRONG **⊕◯◯◯**
Women with non-surgical POI should be informed that ovarian activity may occur. This is associated with a chance of natural conception.	STRONG **⊕◯◯◯**
Women with non-surgical POI should be advised to use contraception if they wish to avoid pregnancy.	STRONG **⊕◯◯◯**

**PICO Question: What fertility interventions are effective?**

**Table:** 

Women with POI should be informed that there are no interventions that have been reliably shown to increase ovarian activity and natural conception rates.	STRONG **⊕⊕⊕◯**
Women with POI should be informed that oocyte donation is an established option to achieve pregnancy after a diagnosis of POI.	STRONG **⊕⊕◯◯**
Women with non-iatrogenic POI and considering assisted reproduction using oocytes donated by their sister should be informed that this includes shared genetic risk and carries a higher risk of ovarian stimulation cycle cancellation.	STRONG **⊕⊕◯◯**

**PICO Question: What therapies are effective for fertility preservation and/or prevention of POI?**

**Table:** 

For iatrogenic causes of POI, fertility preservation can be considered prior to treatment.	CONDITIONAL **⊕⊕◯◯**
The guideline group recommends that fertility preservation is discussed with women at risk of POI. In most women with POI, there is no opportunity for fertility preservation as the follicle pool is depleted.	GPP

**PICO Question: What are the obstetric risks associated with POI?**

**Table:** 

Women should be reassured that natural pregnancies after idiopathic POI or most forms of chemotherapy do not show any higher obstetric or neonatal risk than in the general population.	STRONG **⊕⊕◯◯**
Oocyte donation pregnancies are high risk and should be managed in an appropriate obstetric unit. Women and their partners should be encouraged to disclose the origin of their pregnancy to their obstetric team.	STRONG **⊕⊕◯◯**
Pregnancies occurring after radiation to the uterus are at high risk of obstetric complications and should be managed in an appropriate obstetric unit.	STRONG **⊕⊕◯◯**
Pregnancies in women with Turner Syndrome are at high risk of obstetric and non-obstetric complications and should be managed in an appropriate obstetric unit with cardiologist involvement.	STRONG **⊕⊕◯◯**

**PICO Question: How should fitness for pregnancy be assessed in women with POI?**

**Table:** 

Women presenting for oocyte donation who are suspected of having POI should be investigated for the aetiology of POI prior to oocyte donation.	STRONG **⊕⊕◯◯**
A cardiologist should be involved in care of women considering pregnancy who have received anthracyclines and/or cardiac irradiation.	STRONG **⊕◯◯◯**
Comprehensive cardiac screening and appropriate counselling by both a maternal–fetal medicine specialist and cardiologist with expertise in managing women with Turner Syndrome is recommended prior to planning a pregnancy, especially if oocyte or embryo donation is considered.	STRONG **⊕⊕◯◯**
In addition to usual antenatal screening, women with POI should have their cardiometabolic and thyroid function assessed prior to pregnancy.	STRONG **⊕◯◯◯**
Pregnancy in some women can be of such high risk that HCPs may consider oocyte donation pregnancy to be life threatening and therefore inappropriate.	STRONG **⊕◯◯◯**

**PICO Question: What are the consequences of POI for skeletal health?**

**Table:** 

Women with POI and HCPs should be aware that POI is associated with abnormal bone microarchitecture and reduced bone mineral density.	STRONG **⊕⊕◯◯**
It is suggested that HCPs inform women that POI may be associated with an increased risk of osteoporosis and fracture later in life.	CONDITIONAL **⊕◯◯◯**

**PICO Question: What are the treatment options for bone protection and improvement?**

**Table:** 

Osteoporosis risk factors should be identified and addressed at POI diagnosis and during ongoing care.	STRONG **⊕◯◯◯**
The guideline group recommends that women with POI should be encouraged to adopt a healthy lifestyle (including weight-bearing exercise, healthy diet, avoiding smoking, and maintaining normal body weight) to optimize bone health.	GPP
Dietary supplementation of calcium and vitamin D may be required in women with inadequate vitamin D status and/or calcium intake and may be of benefit in women with low bone mineral density.	CONDITIONAL **⊕⊕◯◯**
HT is recommended to maintain bone density and prevent osteoporosis.	STRONG **⊕⊕◯◯**
A daily dose of hormone replacement therapy (HRT) containing no less than 2 mg oral estradiol or 100 µg transdermal estradiol, or equivalent, is suggested to optimize bone mineral density.	CONDITIONAL **⊕◯◯◯**
Delayed initiation and non-adherence of hormone therapy should be avoided.	STRONG **⊕◯◯◯**
If the combined oral contraceptive is used, then a continuous or extended regimen is recommended to provide continuous estrogen therapy and avoid bone loss.	STRONG **⊕⊕◯◯**
Other pharmacological treatments, including bisphosphonates, should only be considered with advice from an osteoporosis specialist. Particular caution applies to women desiring pregnancy.	STRONG **⊕⊕◯◯**

**PICO Question: How should skeletal health be monitored in women with POI?**

**Table:** 

Where available, measurement of bone mineral density using dual x-ray absorptiometry (DXA) at diagnosis of POI is recommended for all women.	STRONG **⊕⊕◯◯**
If bone mineral density is normal and adequate systemic HT is commenced and adhered to, the value of a repeated DXA scan within 5 years is low.	STRONG **⊕◯◯◯**
Bone mineral density using DXA should be reassessed every 1–3 years, based on individual risk factors, in women with POI who have osteoporosis or low bone density.	STRONG **⊕◯◯◯**
The guideline group recommends that a decrease in bone mineral density should prompt review of HT and potential factors contributing to bone loss. Referral to a specialist may be required.	GPP

**PICO Question: What are the consequences of POI for muscle health?**

**Table:** 

It is suggested that HCPs inform women that POI may be associated with reduced muscle mass, strength, and performance, which may increase the risk of sarcopenia.	CONDITIONAL **⊕⊕◯◯**

**PICO Question: What are the treatment options for muscle protection and improvement?**

**Table:** 

The guideline group recommends that women with POI should be encouraged to adopt a healthy lifestyle (including healthy diet, physical activity, avoiding smoking, and maintaining normal body weight) to aid muscle health.	GPP
HCPs may consider prescribing resistance exercise for women with POI and impaired muscle parameters as resistance exercise increases muscle mass, strength and performance in other populations, although specific evidence in women with POI is lacking.	CONDITIONAL **⊕◯◯◯**
It is suggested that HCPs inform women with POI that HRT prescribed for other indications may also benefit muscle health.	CONDITIONAL **⊕◯◯◯**
The effect of other interventions, including testosterone therapy, on muscle health in women with POI is uncertain and therefore they should not be offered.	STRONG **⊕◯◯◯**

**PICO Question: How should muscle health be monitored in women with POI?**

**Table:** 

The guideline group recommends that HCPs consider screening for sarcopenia at POI diagnosis.	GPP

**PICO Question: What are the consequences of POI for the cardiovascular system?**

**Table:** 

Women with POI should be advised that they are at increased risk of cardiovascular disease, including coronary artery disease, heart failure, and stroke.	STRONG **⊕⊕◯◯**
All women diagnosed with Turner Syndrome should be evaluated by a cardiologist with expertise in congenital heart disease, especially prior to and during pregnancy.	STRONG **⊕⊕◯◯**

**PICO Question: Is estrogen therapy cardio-protective?**

**Table:** 

HCPs and women should be aware that estrogen therapy has beneficial cardiometabolic effects, which can influence cardiovascular disease risk. Non-use of HT is associated with an increased risk of cardiovascular events and mortality, and HT is therefore recommended until the usual age of menopause.	STRONG **⊕⊕◯◯**

**PICO Question: Should cardiovascular risk factors be monitored?**

**Table:** 

The guideline group recommends that cardiovascular risk should be assessed in women diagnosed with POI.	GPP
The guideline group recommends that women with POI should be informed of cardiovascular risk factors that they can modify through lifestyle behavioural change (including avoiding smoking, heart healthy diet, regular physical activity, and maintenance of normal body weight).	GPP
The guideline group recommends that all women with POI should have (at least) annual monitoring of blood pressure, weight, and smoking status.	GPP
The guideline group recommends that all women with POI should have a lipid profile and diabetes screening at diagnosis. Thereafter, frequency of measurement should be based on the presence of hyperlipidaemia, hyperglycaemia, and additional risk factors or global cardiovascular risk.	GPP

**PICO Question: What are the consequences of POI on psychological wellbeing and quality of life?**

**Table:** 

HCPs should be aware that a diagnosis of POI can have a significant impact on psychological wellbeing and quality of life.	STRONG **⊕◯◯◯**
The guideline group recommends offering assessment of psychological health and quality of life to all women with POI.	GPP

**PICO Question: What are the management options for reduced quality of life associated with POI?**

**Table:** 

Personalized care, including psychological support, should be accessible to women with POI.	STRONG **⊕◯◯◯**

**PICO Question: What are the consequences of POI for sexuality?**

**Table:** 

HCPs should advise women that a diagnosis of POI can have a significant impact on sexual wellbeing and function.	STRONG **⊕⊕◯◯**
The guideline group recommends that HCPs routinely and sensitively ask permission of women with POI to discuss sexual wellbeing and function.	GPP

**PICO Question: What are the management options for the effects of POI on sexuality?**

**Table:** 

The guideline group recommends personalized management using the biopsychosocial model for the impact of POI on sexuality.	GPP
Where available, transdermal testosterone therapy, in doses that approximate physiological premenopausal testosterone concentrations, can be considered as it may improve hypoactive sexual desire disorder and sexual function.	CONDITIONAL **⊕⊕◯◯**
HCPs should be aware that HT prescribed to women with POI for other indications may improve sexual function, although the effect is generally small.	STRONG **⊕◯◯◯**

**PICO Question: What treatments are available for genitourinary symptoms in POI?**

**Table:** 

HCPs should offer vaginal estrogen therapy to improve genitourinary and sexual symptoms.	STRONG **⊕◯◯◯**
Women with POI may be offered vaginal estrogen therapy if genitourinary symptoms are not fully relieved by using systemic HT.	CONDITIONAL **⊕◯◯◯**
Vaginal lubricants and moisturizers can be used for treatment of vaginal discomfort and dyspareunia in women with POI and can be combined with other treatments.	CONDITIONAL **⊕◯◯◯**
The guideline group currently does not recommend laser or thermal energy as standard care for genitourinary symptoms due to inconclusive evidence of benefit from RCTs.	GPP

**PICO Question: What are the consequences of POI on cognition/neurological function?**

**Table:** 

HCPs and women should be aware that POI is associated with an increased risk of cognitive impairment and dementia.	STRONG **⊕◯◯◯**
The possible detrimental effect on cognition and increased risk of dementia, parkinsonism, and other neurologic diseases should be discussed when planning bilateral oophorectomy under the age of 45 years, especially for women at an average risk of ovarian cancer.	STRONG **⊕◯◯◯**

**PICO Question: What are the management options for the effect of POI on cognition/neurological function?**

**Table:** 

HT is recommended in women with POI until the usual age of menopause to reduce the possible risk of cognitive impairment and dementia.	STRONG **⊕⊕◯◯**
HT may be recommended in women with POI to protect neurological function even in the absence of menopausal symptoms.	CONDITIONAL **⊕⊕◯◯**
The guideline group recommends that women with POI should be encouraged to adopt a healthy lifestyle (including physical activity, healthy diet, avoiding smoking, and maintaining normal body weight) to reduce the risk of cognitive impairment and dementia.	GPP

**Figure 3.**
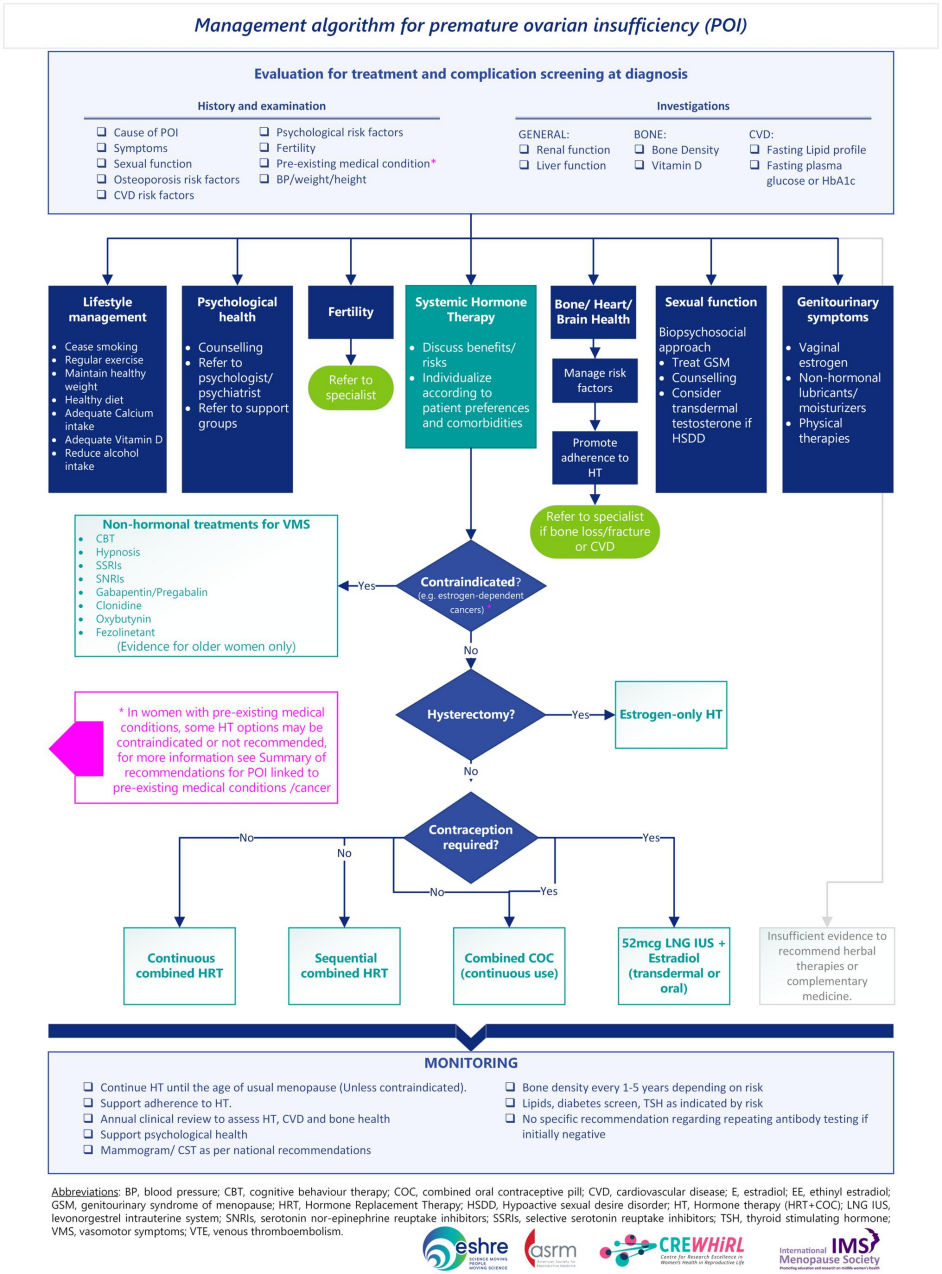
Management algorithm for premature ovarian insufficiency (POI), summarizing the recommendations on evaluation and screening, treatment options, and monitoring.

POI treatment (Fig. 3)

**Hormone therapy (HT) in POI: Principles and indications**

**Table:** 

HT is recommended for women with POI until the usual age of menopause for primary prevention to reduce the risk of morbidity and mortality, whether there are estrogen deficiency symptoms or not.	STRONG **⊕◯◯◯**
Women with POI should be advised that HT is recommended for the treatment of symptoms due to low estrogen concentrations.	STRONG **⊕⊕◯◯**
The guideline group recommends that when women with POI reach the age at which usual menopause occurs, HCPs consider the need for continued HT based on a personalized risk–benefit assessment and current evidence.	GPP
The guideline group recommends that HCPs advise women with POI that hormone replacement therapy (HRT) does not provide contraception, in order to assist them with their family planning	GPP
In women with POI with evidence of intermittent ovarian function and desiring natural pregnancy, recommendations for HRT remain unchanged and do not impact chances of natural conception. A sequential HRT regimen is recommended.	GPP

**PICO Question: What are the risks of HT?**

**Table:** 

Women with POI can be informed that there is no evidence that HT use increases their risk of breast cancer compared to women of the same age without POI.	CONDITIONAL **⊕⊕◯◯**
HT is generally not recommended in women with a history of breast cancer.	STRONG **⊕⊕⊕◯**
Women with BRCA1/2 mutations without a personal history of breast cancer should be advised that HT is an option after risk-reducing bilateral salpingo-oophorectomy.	STRONG **⊕⊕◯◯**
A progestogen should be given in combination with estrogen therapy to all women with an intact uterus to prevent endometrial hyperplasia/cancer.	STRONG **⊕⊕◯◯**
The guideline group recommends that the dose of progestogen is increased when higher doses of estrogen therapy are used.	GPP
The guideline group recommends that in women with POI, as with any women using HT, unscheduled bleeding requires assessment.	GPP
The guideline group recommends that women with POI and a history of endometriosis should be treated with combined estrogen–progestogen HT, even after hysterectomy, to avoid recurrence of endometriosis or malignant transformation.	GPP
Migraine should not be considered a contraindication to HRT use by women with POI.	STRONG **⊕⊕◯◯**
HCPs should consider changing dose, route of administration, or regimen if migraine worsens during HRT.	STRONG **⊕⊕◯◯**
Women with POI and migraine with aura should be advised to use transdermal estrogen as this may be the lowest-risk route of administration.	STRONG **⊕◯◯◯**

**PICO Question: What are the options for HT?**

**Table:** 

The guideline group recommends shared decision making when prescribing each component of HT with consideration of patient preference, contraceptive needs, and presence of co-morbidities.	GPP
Different estrogens/progestogens have variable metabolic and other effects, which should be taken into consideration when personalizing care in POI.	STRONG **⊕⊕◯◯**
The guideline group recommends that HCPs and women should be aware that compounded ‘bio-identical’ preparations of estrogen and progesterone are not recommended due to lack of data regarding efficacy and safety.	GPP
Women with POI should be advised that adherence to HT is important to minimize long-term health risks and therefore long-term follow-up is needed.	STRONG **⊕⊕◯◯**

**Monitoring HT**

**Table:** 

The guideline group recommends that women with POI should have a regular clinical review, addressing individualized risk factors and adherence to therapy.	GPP

**PICO Question: What is the role of testosterone therapy in POI?**

**Table:** 

Testosterone treatment should be considered in women with iatrogenic POI to manage hypoactive sexual desire disorder when other biopsychosocial aetiologies are excluded.	STRONG **⊕⊕◯◯**
Testosterone treatment could be considered in women with non-iatrogenic POI to manage hypoactive sexual desire disorder when other biopsychosocial aetiologies are excluded.	CONDITIONAL **⊕⊕◯◯**
HCPs should be aware that although short-term treatment with transdermal testosterone at doses approximating physiological premenopausal levels is safe, longer term safety data are lacking.	STRONG **⊕⊕◯◯**
The guideline group recommends that women with POI are informed that there are limited data for androgen treatment for indications other than hypoactive sexual desire disorder, and that long-term health effects are unknown.	GPP

**PICO Question: What are the specific considerations for HT in iatrogenic POI?**

**Table:** 

The guideline group recommends a personalized approach to risks and benefits of HT in women with iatrogenic POI after gynaecological/breast cancer.	GPP
HT does not increase the risk of recurrence of squamous cell carcinoma of the cervix and is recommended for women with iatrogenic POI due to the treatment of squamous cell carcinoma.	STRONG **⊕⊕⊕◯**
HT may be associated with a slightly increased risk of recurrence of cervical adenocarcinoma and a personalized approach considering individualized HT risk and benefits is recommended.	STRONG **⊕⊕◯◯**
HCPs could consider HT in women with iatrogenic POI due to early-stage low-risk endometrial adenocarcinoma, as there is no evidence that it increases the risk of cancer recurrence.	CONDITIONAL **⊕⊕◯◯**
HCPs could consider HT in women with iatrogenic POI due to epithelial ovarian cancer.	CONDITIONAL **⊕⊕⊕◯**
The effect of HT on the risk of recurrence of non-epithelial ovarian cancer is uncertain and it is suggested that HCPs use a personalized approach to prescribing HT, including consideration of tumour hormone receptor status.	CONDITIONAL **⊕◯◯◯**
HT should be avoided in women with hormone-dependent ovarian or uterine tumours, including uterine sarcoma, endometrioid carcinoma, ovarian clear cell carcinoma, ovarian granulosa cell tumour, or sex cord-stromal tumours.	STRONG **⊕⊕⊕◯**
Women should be informed of the risks of iatrogenic POI and risks and benefits of HT before bilateral salpingo-oophorectomy to reduce cancer risk (RRSO).	STRONG **⊕◯◯◯**
It is recommended that personalized HT or pubertal induction be commenced in girls/women with POI following haematopoietic stem cell transplantation or other gonadotoxic therapies.	STRONG **⊕⊕◯◯**

**PICO Question: What non-hormonal therapies are available for POI?**

**Table:** 

HCPs could consider non-hormonal pharmacologic and non-pharmacologic therapies for women with POI that are effective in peri-/postmenopausal women, although evidence specific to POI is lacking.	CONDITIONAL **⊕◯◯◯**

**PICO Question: What complementary treatments are effective for managing the sequelae of POI?**

**Table:** 

The guideline group recommends that HCPs should enquire about use of complementary therapies and incorporate individual patient values and preferences into shared decision making about their use.	GPP
Complementary therapies should not be used to replace HT as there is insufficient evidence on their effectiveness for prevention of long-term sequelae of POI.	STRONG **⊕◯◯◯**
Women who are considering the use of Chinese herbal medicine for the management of menopausal symptoms and metabolic risk should be informed that the evidence for benefit is limited but the intervention does not appear to cause significant harm in the short term.	STRONG **⊕◯◯◯**
Women should be informed that there is limited evidence on the effectiveness of acupuncture for menopausal symptoms in POI, and the evidence does not suggest a benefit from adding acupuncture to HT.	STRONG **⊕◯◯◯**
Women who are considering using other nutrient supplements and herbal medicines should be informed that there is insufficient evidence to support their use.	STRONG **⊕◯◯◯**

**PICO Question: What are the lifestyle management options for POI?**

**Table:** 

Women should be aware that a healthy lifestyle, including physical activity, has metabolic and heart benefits in the general population including postmenopausal women, although specific evidence on lifestyle interventions in POI is limited.	STRONG **⊕⊕◯◯**
The guideline group recommends women with POI should be encouraged to adopt a healthy lifestyle to improve their overall well-being and mitigate the risk of potential complications.	GPP

**PICO Question: How should puberty be induced?**

**Table:** 

Puberty should be induced or progressed with estradiol, starting with low dose at the age of 11 years with a gradual increase over 2–3 years.	STRONG **⊕⊕◯◯**
In cases of late diagnosis and for those girls in whom growth is not a concern, HCPs can consider a modified regimen of estradiol therapy.	CONDITIONAL **⊕◯◯◯**
Evidence for the optimum mode of administration (oral or transdermal) is inconclusive. HCPs may prefer transdermal estradiol as it results in more physiological estrogen concentrations.	CONDITIONAL **⊕◯◯◯**
A combined oral contraceptive should not be used for puberty induction.	STRONG **⊕◯◯◯**
The guideline group recommends starting cyclical progestogens after ∼2 years of estrogen therapy or when breakthrough bleeding occurs.	GPP

## Discussion

This paper provides an overview of recommendations for the management of POI, from prevalence, symptoms, diagnosis and causation, to sequelae, monitoring, and treatment. Overall, 145 recommendations have been formulated, 92 supported by research data and 53 good practice points (or statements) based primarily on clinical expertise. The guidelines are based on the best available evidence or, where data of sufficient quality were absent, on recommendations by the guideline group (good practice points).

The current guideline and recommendations are an update of the ESHRE guideline: Management of women with POI, published in 2015/2016 (Webber *et al.*, 2016). The key questions and topics covered in the guideline of 2015/2016 were updated based on the results of a scoping survey, and the evidence supporting the recommendations was updated based on data published between 2015 and 2024, where available.

Of importance are new data indicating a higher prevalence of POI, 3.5–3.7%, than was previously thought (Golezar *et al.*, 2019; Li *et al.*, 2023). This key finding emphasises that POI is not a rare disease, and quite common when the prevalence data for both POI and early menopause (12.2%) are combined, with significant individual and public health implications.

Whilst most of the more recent studies confirm or clarify previous recommendations, almost all guideline questions contain recommendations in which significant changes in clinical practice are to be expected.

One of the key differences relates to the diagnosis of POI, where the 2015/2016 guidance recommended FSH assessments on two occasions to diagnose POI. However, a single FSH assessment in combination with the characteristic clinical picture is now considered sufficient for POI diagnosis, and a second FSH assessment is only required in case of diagnostic uncertainty, such as where the initial FSH level is inconclusive or not in keeping with the clinical picture. This change in guidance should facilitate the rapid and efficient diagnosis of POI, which is particularly important in ensuring prompt commencement of treatment.

Guidance regarding the role of AMH testing in the diagnosis and prediction of POI is also provided. While AMH should not be used as a primary diagnostic test, it may be of value in confirmation of the diagnosis where there is uncertainty, though we should be mindful that it is still not universally available, particularly in primary care.

Recognition of advances in genetic testing is also included with a recommendation regarding next-generation sequencing where available. Although access to such testing currently varies between countries and regions, it is important that we strive to determine the aetiology of POI where possible as this may help to personalize individual and familial risks, particularly when linked to genes with specific implications for fertility and malignancy.

A new recommendation was introduced regarding care for women at the time of diagnosis, emphasising the psychological impact that diagnosis can have and the importance of sensitively conveying the diagnosis and shared decision making.

Emerging data indicate that changes in muscle parameters associated with POI occur, and thus a topic on muscle health was included. More research is urgently required in this area.

A recommendation regarding the frequency of bone densitometry (DXA) (where available) to monitor osteopenia and osteoporosis in women with POI was also an important change from the previous version as this should facilitate the management of one of the most common and troublesome long-term problems associated with POI. However, the value of repeated DXA monitoring in women with normal bone density remains uncertain.

The updated guideline again emphasises the importance of HT for symptom relief and prevention of chronic diseases in women with POI. However, it extends the 2015/2016 guideline by including recommendations regarding estrogen doses and regimens and continuous use of the combined oral contraceptive.

Recommendations regarding testosterone therapy have also been updated, reflecting new evidence and a consensus statement regarding women at usual age of menopause, although further research in women with POI is still needed (Davis *et al.*, 2019).

Although data specific to POI populations are lacking, recommendations regarding the use of non-pharmacological therapies for menopausal symptoms, lifestyle management, and complementary therapies are included, mainly extrapolated from women at usual age of menopause. Non-hormonal pharmacological therapies recommended for menopausal women with vasomotor symptoms are likely to be effective in POI. Healthy lifestyle behaviours will benefit women with POI and should underpin all recommended interventions. Complementary therapies should not be used instead of HT because of limited evidence regarding efficacy, particularly for the long-term health sequelae of POI.

Induction of puberty is now recommended from age 11 years with emphasis on the use of estradiol to optimize metabolic benefits, uterine, and breast development, rather than conjugated equine estrogens or ethinylestradiol.

The literature searches not only resulted in recommendations being formulated but also highlighted a number of areas where the evidence was too scarce to formulate clear and strong recommendations. Of the evidence-based recommendations, almost 76% were formulated as strong recommendations (i.e. appropriate for most women with POI), even if the evidence base was limited to observational data (level very low or low), supporting a call for ongoing and future research.

Hence, the guideline group concluded that there is still an urgent need for more research on the most appropriate diagnostic and treatment options, but also to further elaborate the impact of estrogen deficiency on the health and life expectancy of the women diagnosed with POI. This guideline provides 30 recommendations for research, intended to inspire researchers, and hopefully also to facilitate funding for studies in POI (Supplementary File S1).

In summary, the 2024 Guideline on POI is a comprehensive update of the existing evidence and should assist healthcare professionals in the care of women with POI. Active involvement and input by patient representatives at all stages was central to the success of this endeavour. The detailed guideline document can be accessed via the societies’ websites (e.g. www.eshre.eu/guidelines). In order to maximize uptake of the guideline, plans for dissemination and translation to complement the guideline are currently being deployed.

## Supplementary Material

hoae065_Supplementary_Data

## Data Availability

The full guideline and supporting data (literature report, evidence tables) are available on www.eshre.eu/guidelines.

## Appendix

**Collaborators:**

Ladan Yeganeh, Monash Centre for Health Research and Implementation, Monash University, Melbourne, VIC, Australia.

Rinky Giri, Endocrinology Unit, Monash Health, Melbourne, VIC, Australia; Reproductive Biology Unit, Monash Health, Melbourne, VIC, Australia; Hudson Institute of Medical Research, Melbourne, VIC, Australia.

Madeline Flanagan, Department of Obstetrics and Gynaecology, Monash University, Melbourne, VIC, Australia
