# Supplementary material for: Evidence-based guideline: premature ovarian insufficiency,
Source: Hum Reprod Open. 2024 Dec 9;2024(4):hoae065. doi: 10.1093/hropen/hoae065 (PMC11631070; doi:10.1093/hropen/hoae065)
Supplement: hoae065_Supplementary_Data [file hoae065_supplementary_data.docx]

**Supplementary File S1: List of research recommendations.**

| Risk factor, diagnosis and causation | |
| --- | --- |
|  | - Further research is required to clarify ethnic and geographic variation in POI prevalence to inform future potential screening and public health intervention strategies - Further research is required to (i) identify and clarify risk factors for POI, in addition to those related to early menopause, especially the role of family history (e.g. mother’s age at natural menopause), socio-economic factors, lifestyle and environmental chemicals; and to (ii) identify and quantify strategies that may mitigate modifiable risk factors. - Further research is required to establish the optimal FSH criteria for the diagnosis of POI and a sensitive and specific alternative biomarker that is readily available. - Further research is required into the value of AMH as a predictive or diagnostic test for POI. - Ongoing research both in animal models and humans is required to identify additional genes involved in POI and to allow uncovering of molecular defects in non-coding regions of known genes, copy number variations and structural variations. - Research to identify new genes can lead to a better understanding of ovarian physiology and pave the way for developing new care strategies and treatments. POI registries can advance such research. - Exploration of how genetic variants combine with environmental factors to determine the clinical phenotype is also needed. This will markedly enhance the positivity of genetic testing, availability of genetic testing and development of novel management strategies. - Improvements in genetic sequencing techniques and interpretive approaches may provide a more precise determination of the mechanisms underlying ovarian dysfunction, and facilitate screening, diagnosis, and cost-effectiveness. - Research into methods for reliable prediction of POI and monitoring of ovarian function in relatives of women with non-iatrogenic POI is needed. - Further research into the outcomes of fertility preservation in the specific group of women with a family history of POI is indicated. |

| Sequelae of POI and treatments | |
| --- | --- |
| Musculoskeletal health | - Further research in bone and muscle health in POI is required to   (i) clarify fracture risk associated with POI and the effect of hormone therapy (HT) on this outcome;  (ii) determine the optimal regimen of HT for prevention of osteoporosis and whether HT regimens need to change across the life course;  (iii) determine the best strategies for monitoring of bone health including screening interval, role of bone turnover markers and newer imaging modalities;  (iv) clarify the changes in muscle mass and function associated with POI; (v) investigate the effect of nutritional supplements (such as protein or Vitamin K) and exercise on muscle parameters, bone density and fracture in women with POI;  (vi) clarify the role of bone specific agents in managing POI associated osteoporosis;  (vii) identify strategies for assessment and monitoring of muscle health in this population including defining sarcopenia; and  (viii) examine the role of HT and other strategies to maintain muscle health. |
| Cardiovascular health | - There is a need for long-term randomized prospective studies to determine the optimal routes, doses, and regimens of HT and particularly their impact on quality of life, fertility, bone, cardiovascular, cognitive health and life expectancy. - The long-term impact on risk factors such as breast cancer, VTE and stroke should also be investigated. |
| Quality of Life | - QoL research is needed involving prospective studies with the use of comprehensive scale validated in women with spontaneous and iatrogenic POI. - The role of medical and psychological interventions in improving QoL should be implemented with the aid of adequate instruments developed in collaboration with women with POI of different aetiologies. - Studies conducted in a multidimensional perspective are needed to assess psychosexual and psychosocial changes in women with POI and the entity of distress. - A process of care specifically developed for women with POI presenting sexual symptoms is warranted. |
| Sexuality | - A better understanding on the effects of different type and dose of systemic estrogens alone or in combination with specific progestogens on sexuality of POI is warranted. - Studies should evaluate the safety of testosterone when applied for a longer period (more than 6 months) to improve sexual function in POI. - Studies should evaluate the efficacy and safety of testosterone treatment on several domains of health in women with POI. - More research is needed to understand the difference between iatrogenic and non-iatrogenic POI in terms of testosterone levels and testosterone treatments. |
| Genitourinary symptoms | - More research conducted specifically in women with POI is needed on hormonal approaches for genitourinary symptoms. - Studies should explore the efficacy and safety of laser therapy and other non-hormonal approaches to relief genitourinary symptoms in women with POI, especially in those with contraindications to vaginal estrogen. |
| Neurological function | - Research is needed to further clarify the pathogenetic mechanisms mediating the effects of POI, both non-iatrogenic and iatrogenic, on adverse neurological outcomes including cognitive decline and dementia. - Further research is needed to confirm the effects of Hormone Replacement Therapy (HRT) on brain ageing in women who underwent POI, both with and without menopausal symptoms. |
| HT | - Investigating the benefits/ risks of HT continuing for a further 5 years or more after the age of usual menopause |
| Lifestyle | - Due to limited evidence available for POI, ongoing research is essential to explore the specific effect of lifestyle interventions on the features of menopause, QoL and cardiovascular outcomes for women with this condition. |
| Puberty induction | - Research concerning the optimal age for induction of puberty is still needed, with increased focus on cognitive function, sexual function, uterine development, cardiovascular status, development of a normal body composition including bone acquisition and other areas. - Likewise, in induction of puberty, there is a need to establish the optimal route of delivery of first estradiol at escalating doses and then progesterone, when sequential therapy is needed. - Establishing the long-term outcome of appropriate puberty induction using both a clinical and an epidemiological approach is also needed. - The fundamental understanding of why POI develops in conditions like Turner syndrome remains an enigma and should also be investigated. |
